# Imagerie multimodale d’un nævus choroïdien atypique

**DOI:** 10.11604/pamj.2020.37.256.25843

**Published:** 2020-11-19

**Authors:** Ahmed Mahjoub, Safa Hadj Salah, Tesnim Mhamdi, Nadia Ben Abdesslem, Mohamed Ghorbel, Hachemi Mahjoub

**Affiliations:** 1 Service d’Ophtalmologie, Centre Hospitalier Universitaire Farhat Hached de Sousse, Sousse, Tunisie

**Keywords:** Nævus choroïdien, néo-vaisseau choroïdien, imagerie rétinienne, Choroidal nevus, choroidal neo-vessel, multimodal imaging

## Abstract

Les nævi choroïdiens sont des lésions fréquentes, asymptomatiques, de diagnostic généralement aisé. Ils peuvent entraîner un syndrome maculaire en cas de dégénérescence ou de néo vascularisation choroïdienne. En cas de doute, la combinaison des différents moyens d'investigations que sont l'examen clinique, l'échographie, l'angiographie à la fluorescéine et le vert d'indocyanine (ICG) permet de redresser le diagnostic. Nous rapportons le cas d´une patiente qui a présenté une lésion choroïdienne atypique, et chez qui l´imagerie multimodale a permis de retenir le diagnostic d'un nævus choroïdien achrome compliqué de néo vascularisation choroïdienne.

## Abstract (en)

Choroidal nevi are frequent, asymptomatic, usually easy-to-diagnose lesions. They can cause macular syndrome in patients with choridal degeneration or neovascularization. In doubtful cases, the combination of different diagnostic means, such as clinical examination, ultrasound, fluorescein angiography and indocyanine green (ICG) can help to correct the diagnosis. We here report the case of a female patient with atypical choroidal lesion, in whom multimodal imaging allowed to retain the diagnosis of achromic choroidal nevus complicated by choroidal neovascularization.

## Introduction

Les tumeurs de la choroïde sont multiples: bénignes ou malignes, primitives ou secondaires. La multiplicité de ces lésions ainsi que leur polymorphisme clinique expliquent les difficultés et les erreurs diagnostiques. Nous rapportons le cas d´une patiente qui a présenté une lésion choroïdienne atypique, et chez qui l´imagerie multimodale a permis de redresser le diagnostic.

## Patient et observation

**Figure 1:**
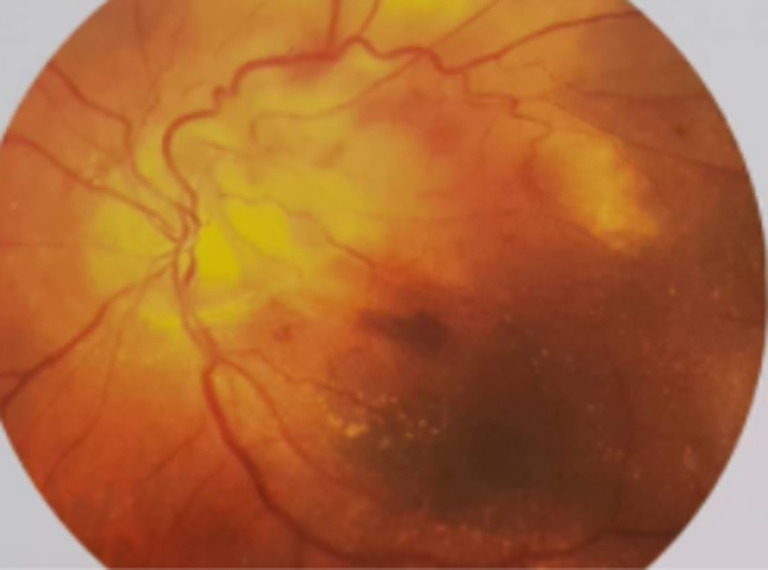
lésion juxta papillaire avec hémorragies et exsudats autour

**Figure 2:**
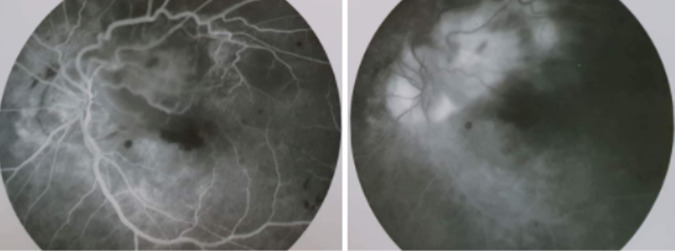
angiographie à la fluorescéine; hyper fluorescence au temps précoces avec imprégnation tardive

**Figure 3:**
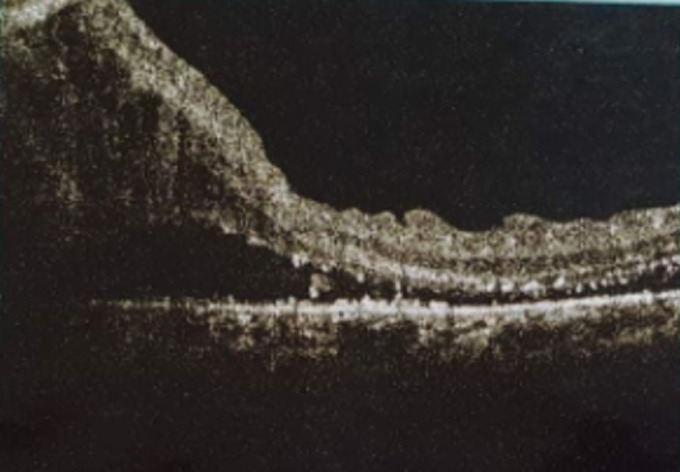
OCT passant par la lésion; la lésion n´est pas visible, on note un épaississement rétinien et un DSR en regard de la lésion

**Figure 4:**
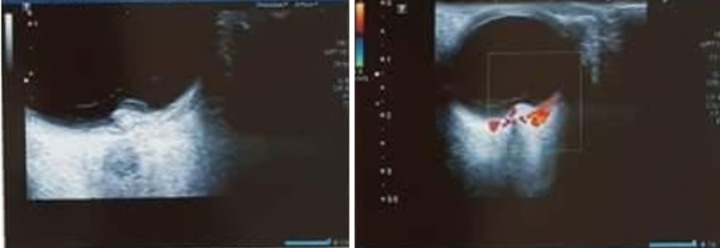
écho doppler oculaire; lésion hyperéchogène fusiforme non vascularisée faisant 5 millimètres, avec DSR en regard, sans excavation choroïdienne

Il s´agit d´une patiente âgée de 65 ans, sans antécédents pathologiques notables, qui se présente pour une baisse progressive de la vision de l´œil gauche évoluant depuis 9 mois. L´examen de l´œil gauche montre une acuité visuelle limitée aux perceptions lumineuses. Le fond d´œil retrouve une lésion du pôle postérieur juxta papillaire, jaunâtre avec des hémorragies intra rétiniennes et des exsudats autour et un décollement séreux rétiniens (Figure 1). L´angiographie à la fluorescéine montre une hypo fluorescence précoce de la lésion avec imprégnation tardive (Figure 2). La tomographie par cohérence optique (OCT) maculaire confirme le décollement séreux rétinien (DSR), mais ne montre pas la lésion (Figure 3). A l´écho Doppler oculaire, la lésion faisait 5 millimètres et était non vascularisée (Figure 4). A ce stade, les diagnostics évoqués étaient un hémangiome choroïdien, un nævus choroïdien atypique, une métastase choroïdienne et un mélanome choroïdien.

**Figure 5:**
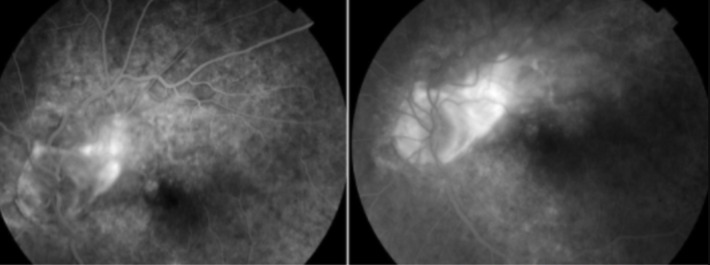
angiographie à la fluorescéine; effet fenêtre péri papillaire

**Figure 6:**
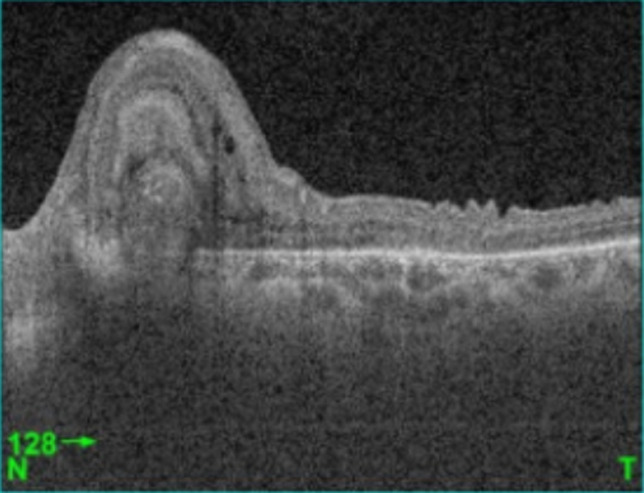
fibrose rétinienne en regard de la lésion et membrane épi rétinienne

**Figure 7:**
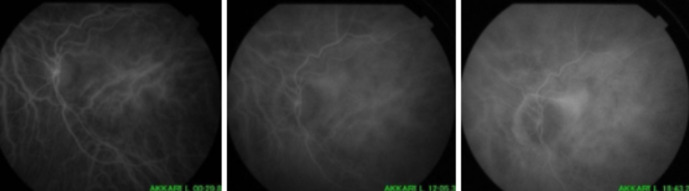
angiographie au vert d´indocyanine; la lésion est iso fluorescente aux temps précoces et tardifs de la séquence angiographique

L´angiographie au vert d'indocyanine (ICG) n´a pas pu être réalisée à ce stade par défaut du produit de contraste. L´évolution spontanée après 2 mois s´est faite vers la régression de la symptomatologie. Au fond d´œil de contrôle on a noté une fibrose rétinienne. L´angiographie à la fluorescéine a montré un effet fenêtre péri papillaire (Figure 5), et l´OCT de contrôle a montré une fibrose rétinienne et a noté la disparition du DSR (Figure 6). Une angiographie à l´ICG réalisée à ce stade a montré que la lésion est iso fluorescente aux temps précoces et tardifs, sans diffusion (Figure 7). La combinaison de l´imagerie multimodale a permis de conclure au diagnostic de nævus choroïdien achrome compliquée d´une néo vascularisation choroïdienne.

## Discussion

Les nævi choroïdiens sont des lésions fréquentes, on estime que 6,5% des patients en sont porteurs [1]. Ils sont généralement asymptomatiques, découverts lors d´un examen systématique du fond d´œil, mais ils peuvent entraîner rarement un syndrome maculaire par néo vascularisation choroïdienne, ou en cas de dégénérescence. A l´examen, il s´agit de lésions planes, d´épaisseur inférieure à 1,5mm, à bords flous avec un diamètre inférieur à 6mm. La pigmentation est variable; 13% des nævi sont achromes. Ils présentent souvent des druses en surface qui témoignent de la chronicité de ces lésions. Classiquement, ils ne présentent pas de pigment orange en surface ni de décollement séreux.

La plupart de ces lésions ne posent aucun problème diagnostique; il existe cependant une catégorie de lésions de la choroïde pour lesquelles le diagnostic est plus difficile et nécessite le recours à une imagerie multimodale. Les facteurs d´atypie retrouvés sont l'épaisseur tumorale, l'atteinte de la papille, la présence de troubles visuels, de pigment orange et d'un décollement séreux, et la présence de pin points à l´angiographie [2, 3]. A l´angiographie à l´ICG, le nævus pigmenté apparaît hypo fluorescent durant toutes les phases de l'ICG (précoces, intermédiaires et tardives). Quand il s'agit d'un nævus achrome, les vaisseaux choroïdiens intrinsèques sont plus apparents et le nævus apparaît hyper- ou iso fluorescent dans les phases précoces et intermédiaires, il peut le rester au temps tardif ou devenir hypo fluorescent [4].

## Conclusion

Le nævus choroïdien est une lésion fréquente, asymptomatique, de diagnostic généralement aisé. Cependant, en cas de dégénérescence ou de néo vascularisation choroïdienne, le diagnostic est plus difficile, d´où l'importance de la combinaison des différents moyens d'investigations que sont l'examen clinique, l'échographie, l'angiographie à la fluorescéine et l'ICG. Dans tous les cas, le diagnostic d´un nævus choroïdien impose une surveillance ophtalmologique régulière afin de détecter précocement une éventuelle croissance tumorale.
